# Visual Impairment and Driving in Older Adults: A Narrative Review

**DOI:** 10.1007/s11940-026-00871-z

**Published:** 2026-05-22

**Authors:** Shu Xu, Joshua R. Ehrlich, Lindsey B. De Lott

**Affiliations:** 1 Institute for Social Research, University of Michigan, Ann Arbor, MI USA; 2 Michigan Sensory Health and Aging Research Collaborative (Mi-SHARC), University of Michigan, Ann Arbor, MI USA; 3 Ophthalmology and Visual Sciences, University of Michigan, Ann Arbor, MI USA

**Keywords:** Visual impairment, Driving safety, Driving performance, Older adults, Visual field loss, Contrast sensitivity

## Abstract

**Purpose of Review:**

This narrative review synthesizes current evidence on the relationship between visual impairment and driving safety in older adults, with a focus on the visual functions required for safe driving, the impact of common visual deficits, and current approaches to clinical evaluation and management.

**Recent Findings:**

Recent studies have strengthened evidence that visual functions beyond high contrast visual acuity--particularly visual field sensitivity, contrast sensitivity, and visual processing speed--are associated with driving performance and crash risk. Emerging studies also highlight the role of higher-order visuospatial processing and the integration of visual input with cognitive, multisensory, and motor systems. Advances in functional assessment tools and in-vehicle technologies, such as advanced driver-assistance systems, may further enhance evaluation and support driving safety.

**Summary:**

Driving safety in older adults with visual impairment is multifactorial and cannot be adequately assessed by a single visual measure. A multidomain approach incorporating visual, cognitive, and functional assessment is essential for clinical decision-making. Management should emphasize treatment of reversible conditions, patient-centered counseling, and referral for functional driving evaluation when appropriate. Future research should focus on developing functionally relevant, real-world measures to better guide clinical practice and policy.

**Opinion Statement:**

Driving is a visually intensive activity that plays a central role in maintaining independence, mobility, and social participation for older adults. As global populations age, the number of older drivers is increasing, making safe mobility an important public health concern. Visual impairment is common in later life and may arise from both ocular disease and neurologic conditions affecting the visual pathways or higher-order visual processing. Although driver licensing systems worldwide rely heavily on visual acuity thresholds to determine visual fitness to drive, growing evidence indicates that visual acuity alone provides an incomplete assessment of driving safety. Safe driving depends on the coordinated function of multiple visual domains, including central vision, peripheral vision, contrast sensitivity, motion and depth perception, visual processing speed, and higher-order visuospatial processing. Impairments in these domains may compromise hazard detection, situational awareness, and timely responses to changing traffic conditions, even when central visual acuity remains relatively preserved. In addition, driving performance requires integration of visual input with cognitive, multisensory, and motor systems, which may be affected by neurologic disease and age-related changes in sensory processing. Clinical evaluation of older drivers with visual impairment should therefore extend beyond visual acuity to incorporate multidomain assessment of visual and neurologic function when concerns arise. Management strategies should prioritize treatment of reversible visual conditions, patient counseling regarding adaptive driving behaviors, and referral for functional driving evaluation when appropriate. Ultimately, decisions regarding driving must balance preservation of independence with public safety. Continued research is needed to develop evidence-based screening strategies and policies that better reflect the complex visual demands of real-world driving.

## Introduction

### Why Driving Matters in Older Adults

Driving represents a critical component of independence and mobility for older adults, enabling access to medical care, social engagement, daily activities, and supporting overall quality of life [1, 2, 3]. Conversely, driving cessation is associated with adverse outcomes, including increased risk of depression, social isolation, and reduced community participation [1, 4]. The impact of driving cessation likely varies across geographic and cultural contexts; for example, older adults in much of the U.S. face greater challenges when they are no longer able to drive due to car-dependent infrastructure and limited public transportation [5].

Population aging further underscores the importance of safe mobility. The global population aged ≥ 65 years is projected to increase from 761 million in 2021 to 1.6 billion by 2050 [6]. As populations age, the number of older drivers and miles traveled continue to increase, making driving safety among older adults an increasingly important public health issue. In the U.S., although fatal motor vehicle crashes per licensed driver have declined since the mid-1990s, older adults remain more vulnerable to serious injury or death due to age-related fragility [7].

### Visual Impairment and Driving

Vision is the primary sensory modality for driving, supporting hazard detection, lane maintenance, and navigation in dynamic environments. Visual impairment is common worldwide and increases substantially with advancing age. Globally, at least 2.2 billion people are estimated to have visual impairment [8]. In the U.S., more than one-quarter of adults aged 71 years and older have some form of visual impairment [9], and the number of affected individuals is expected to increase as populations age.

Driving performance depends on the integration of multiple visual functions, including visual acuity, peripheral vision, contrast sensitivity, motion perception, depth perception, and rapid visual processing [10]. These functions decline with age and may be further affected by common ocular diseases such as cataract, age-related macular degeneration, and glaucoma [11, 12]. However, legal vision requirements rely heavily on a single test of visual function - visual acuity- and requirements vary widely both in the US and globally. As a result, many older adults meet legal requirements for driving in their communities but experience visual function limitation that impair driving performance.

**Fig. 1:**
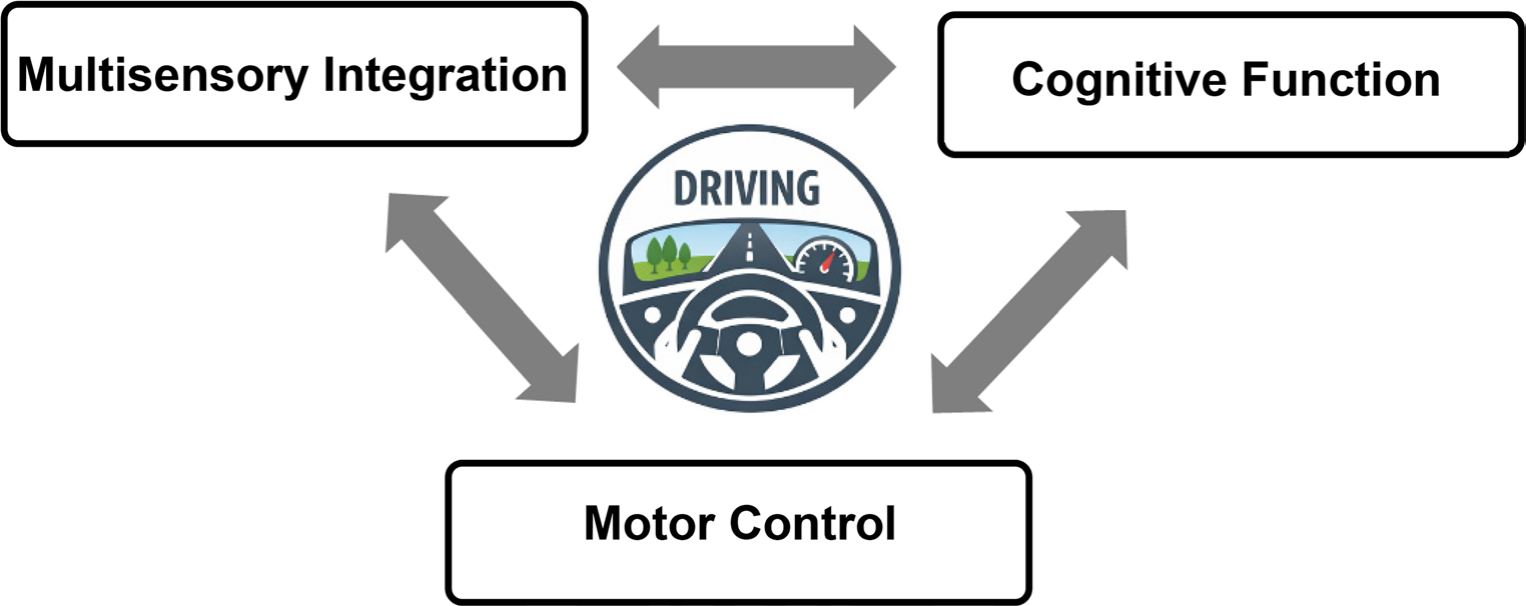
Conceptual model of pathways linking visual impairment to driving risk in older adults

Safe driving also requires integration of visual information with multisensory, cognitive and motor systems [13] (Fig. 1). Neurologic disorders, such as neurodegenerative diseases, affecting higher-order visual processing may therefore impair driving performance [14] even when standard measures of visual function are intact, such as visual acuity and contrast sensitivity [14, 15, 16, 17]. In addition, visual impairment itself is a risk factor for dementia [18], which may further affect the complex sensory and cognitive processes required for safe driving.

### Purpose of Review

Foundational reviews have previously described the relationship between vision and driving [10]. However, a growing body of research has expanded understanding of how specific visual impairments, higher-order visual processing, and real-world driving behavior interact to influence driving safety in older adults in the past decade. This narrative review synthesizes our current understanding of how visual impairment affects driving performance and safety in older adults. We outline the visual functions required for driving, review common types of visual dysfunction and their impact on driving outcomes, and discuss clinical approaches to assessment and management. We also highlight emerging technologies that may support safe driving. This review aims to provide clinicians with a practical framework for understanding the complex relationship between driving and vision impairment while balancing patient autonomy with public safety considerations.

## Visual Demands of Driving: What Visual Abilities Does Driving Require?

Driving is a complex visuomotor task that requires continuous interpretation of dynamic environmental information. Unlike standard clinical vision tests performed under controlled conditions, real-world driving involves rapidly changing stimuli under varying levels of complexity, illumination, and traffic density. Safe driving therefore depends on multiple visual domains working together, including central and peripheral vision, visual search, contrast sensitivity, motion and depth perception, and rapid visual processing [10]. Impairment in any of these domains may compromise hazard detection, decision-making, and timely responses to changing road conditions.

*Central vision* is essential for high-resolution tasks such as reading road signs, recognizing traffic signals, and identifying hazards directly ahead [19]. *Peripheral vision* supports situational awareness by enabling detection of surrounding vehicles, pedestrians, and hazards emerging from outside central fixation. Together, central and peripheral vision allow drivers to maintain lane position while monitoring the broader traffic environment [10, 20]. Driving also requires efficient visual search and hazard detection. Coordinated eye and head movements are needed to scan mirrors, monitor intersections, and identify emerging risks. These processes are closely linked to motion perception and the ability to estimate the speed and trajectory of surrounding objects, which together support anticipation of hazards and timely responses in dynamic traffic environments [21, 22]. Contrast sensitivity is particularly important under low-visibility conditions such as nighttime, glare, or adverse weather, where hazards may not clearly stand out from their background [23, 24]. Depth perception supports judgment of distance and spatial relationships and is especially relevant for lane changes, merging, and maintaining safe following distances [25]. In addition, visual processing speed determines how quickly visual information can be recognized and translated into appropriate action; slowed processing may delay hazard recognition and increase reaction time [26, 27].

### Integration of Visual Information with Multisensory, Cognitive, and Motor Systems.

Although vision provides the primary sensory input during driving, safe vehicle operation depends on the integration of visual information with other sensory, cognitive, and motor systems. Multisensory integration plays a fundamental role in perception of self-motion and spatial orientation during mobility tasks. Perception of self-motion during driving relies on neural integration of visual optic flow with vestibular and proprioceptive signals that encode head motion and acceleration, enabling drivers to estimate vehicle movement and heading direction and maintain spatial orientation [28, 29]. Auditory cues also contribute to situational awareness during driving. Sounds from approaching vehicles, emergency sirens, or horns may alert drivers to hazards that are not yet visible or are located outside the field of view. These signals can prompt drivers to shift visual attention and initiate compensatory scanning behaviors. In visually complex environments, auditory information may therefore support earlier recognition of potential hazards and enhance overall hazard detection [30]. Although evidence is limited and findings are inconsistent [31], the role of hearing loss in driving safety has received increasing attention and some studies suggest an association between hearing impairment and motor vehicle crash risk [32]. These findings raise the possibility that driving risk may be influenced not only by vision loss, but also by reduced integration with other sensory inputs. Combined multisensory deficits, such as concurrent vision and hearing impairments, may further increase this risk [33].

In addition to sensory processing, safe driving requires higher-order cognitive functions that coordinate sensory input with decision-making and motor responses. Effective driving requires the ability to divide attention among multiple stimuli, prioritize relevant environmental cues, and anticipate potential hazards. Impairments in attention, visuospatial processing, or executive function may therefore interfere with the ability to interpret visual information and anticipate risks during driving [10, 16].

Motor systems translate sensory perception and cognitive decisions into physical actions required for vehicle control. Drivers must coordinate eye, head, and limb movements to maintain lane position, adjust speed, and respond to unexpected events. When visual input is degraded, effective coordination between sensory processing, cognitive control, and motor responses becomes increasingly important for maintaining safe driving performance. Age-related changes in sensory processing and cognitive function may alter these integrated systems. Older adults may rely more heavily on visual information during self-motion perception, even when visual cues are unreliable or conflict with vestibular signals. Such changes in sensory weighting may influence spatial orientation and mobility performance, potentially affecting driving safety [28]. Neurologic conditions that affect visuospatial processing or attention may further disrupt these processes, compromising driving performance even when basic visual acuity remains relatively preserved [16].

## Types of Visual Impairment Relevant to Driving

Driving performance can be affected by impairments across multiple visual domains (Table 1). The following sections summarize common types of visual dysfunction and their potential impact on driving.

**Table 1:** Visual functions relevant to driving safety in older adults

Visual Impairment	Common causes in older adults	Functional impact on driving	Screening tools (examples)
Reduced high contrast visual acuity	Age-related macular degeneration (AMD), cataracts, glaucoma, diabetic retinopathy (DR), uncorrected refractive error.	Reduced ability to identify distant objects at distance and near such as road signs, traffic signals, and pedestrians	Snellen chart, near card
Visual field loss	Glaucoma, DR, stroke, traumatic brain injury	Impaired detection of hazards emerging from peripheral areas; difficulty monitoring surrounding traffic	Automated perimetry (Humphrey, Esterman visual field test)
Contrast sensitivity impairment	Cataract, glaucoma, DR, AMD, Optic neuropathies	Difficulty detecting low-contrast hazards, night-driving difficulty, glare from headlights	Pelli–Robson contrast sensitivity chart
Ocular alignment and Diplopia	Cranial nerve palsies, thyroid eye disease, decompensated strabismus, myasthenia gravis	Diplopia, impaired depth perception, visual confusion	Cover test, prism testing, binocular vision assessment
Visuospatial dysfunction	Neurodegeneration, Parkinson’s disease, stroke.	Impaired spatial attention, hazard detection, navigation, and visual scene interpretation	UFOV, Trail Making Test, clock drawing test

### Reduced High-Contrast Visual Acuity

High-contrast visual acuity is typically measured using standardized charts (e.g., the Snellen chart) under optimal lighting conditions and reflects the ability to resolve fine spatial detail. Reduced visual acuity can limit the ability to identify distant objects such as road signs, traffic signals, and pedestrians [24]. Visual acuity is the most commonly assessed visual function in driver licensing regulations worldwide, with most jurisdictions requiring a minimum acuity of approximately 20/40 (Snellen notation in feet equivalent to 6/12 in metric notation) in the better eye for an unrestricted private driver’s license [10, 20, 34].

Despite its widespread use, visual acuity alone is a relatively poor predictor of crash risk compared with other visual functions such as visual field, contrast sensitivity, and processing speed [10]. Similar findings have been reported in recent systematic reviews, which show modest or inconsistent associations between acuity impairment and crash involvement among private drivers [35, 36]. One explanation is that standard visual acuity testing measures static high-contrast letter recognition, which differs from the dynamic visual demands of real-world driving. Driving requires continuous monitoring of complex environments, detection of hazards across the visual field, and rapid interpretation of visual information. Consequently, impairments in other visual domains may have greater impact on driving safety than reductions in central acuity alone. Nevertheless, reduced acuity may contribute to difficulties with object identification and is independently associated with increased driving avoidance and cessation in older adults [37].

### Visual Field Loss

Visual field loss is one of the most consistently studied visual impairments in relation to driving safety. Peripheral vision is often tested both clinically and for research purposes using automated perimetry or, more recently, validated digital visual field applications. The normal visual field is approximately 160 degrees in the horizontal plane and 130 degrees in the vertical plane. Intact peripheral vision is important for detection of hazards outside of the central visual field and is essential for monitoring surrounding traffic, maintaining lane position, and detecting pedestrians or vehicles approaching from the sides [38]. Visual field loss may arise from both ocular and neurologic causes. Common ocular conditions include glaucoma, which produces progressive peripheral field loss [39], whereas neurologic disorders such as stroke or traumatic brain injury involving the optic chiasm, optic radiations, or occipital cortex may cause a bitemporal hemianopia, homonymous hemianopia, or quadrantanopia [40, 41]. In most cases these deficits are static, resulting from structural damage to the retina, optic nerve, optic chiasm, optic radiations, or occipital cortex (i.e., primary visual cortex), although transient visual field disturbances may occur in conditions such as migraine aura, seizure, or transient ischemic attacks [42]. Unlike chronic visual field loss, these episodic disturbances temporarily impair spatial perception but are intermittent and best treated by trying to prevent recurrences [43].

Studies have reported associations between visual field impairment and increased crash risk. A landmark population-based study by Owsley et al. (1998) demonstrated that reduced visual field sensitivity, measured using automated perimetry as sensitivity to light stimuli across the visual field, was associated with higher crash involvement among older drivers [26]. Similarly, studies of drivers with glaucoma have reported increased rates of crashes among those with moderate or severe visual field impairment in the worse-functioning eye, independent of visual acuity, suggesting that the observed association is more likely attributable to visual field loss rather than differences in central acuity [44]. A systematic review examining driving performance in individuals with homonymous hemianopias and quadrantanopia found that visual field loss can impair hazard detection, lane positioning, and scanning behavior during both simulator and on-road driving assessments [45]. Longitudinal and naturalistic driving studies further demonstrate that greater visual field loss is associated with higher rates of at-fault crashes and near-crash events [27, 35, 46]. Additionally, reductions in binocular visual field sensitivity have been associated with increased safety-critical events, including sudden braking and near-collisions [47, 48]. However, functional impact varies across individuals, as some drivers develop compensatory scanning strategies into the impaired visual field, which may partially mitigate the effects of field loss [41, 45, 47].

### Contrast Sensitivity and Glare Sensitivity

Contrast sensitivity refers to the ability to detect objects that differ only slightly in luminance from their background, while glare sensitivity describes reduced visual performance caused by bright light sources that degrade retinal image contrast [49]. These functions are particularly relevant for driving because many hazards appear under low-contrast conditions, such as pedestrians wearing dark clothing, poorly illuminated road markings, or obstacles in dim lighting [24]. Impairments in contrast sensitivity and glare tolerance commonly occur in ocular diseases affecting older adults, including cataract, age-related macular degeneration, glaucoma, and diabetic retinopathy. Cataract frequently impairs contrast sensitivity and increases glare sensitivity even when visual acuity remains relatively preserved. Optic neuropathies, such as optic neuritis or ischemic optic neuropathy, may also impair contrast perception through damage to the retinal ganglion cells [50].

Evidence suggests that contrast sensitivity may be more strongly associated with driving performance than visual acuity alone. Reduced contrast sensitivity has been linked to delayed hazard detection, poorer sign recognition, and impaired lane-keeping, particularly under low-illumination conditions [51]. In a cohort of older drivers with prevalent age-related eye disease, impaired contrast sensitivity was associated with substantially higher rates of at-fault crashes and near-crashes, with rate ratios approaching 2.7 when objective mileage-based exposure was considered. In this study, only 3 participants had impaired visual acuity, suggesting that contrast sensitivity may capture driving-relevant visual dysfunction not reflected by standard high-contrast acuity measures [27].

Lighting conditions appear to play an important role in this relationship, with measures obtained under lower illumination showing stronger associations with higher rates of both overall and at-fault crashes than those measured under standard clinical lighting [35, 49]. Glare sensitivity may further exacerbate these difficulties. Age-related changes in the crystalline lens increase intraocular light scatter, making older drivers more susceptible to glare from oncoming headlights or sunlight. Cataract is a well-recognized cause of glare disability, and treatment with cataract surgery has been associated with reduced motor vehicle crash risk in several studies [35].

### Ocular Misalignment and Diplopia

Ocular misalignment (strabismus) and diplopia represent binocular vision impairments that may disrupt driving by producing double images, visual confusion, and impaired spatial perception [52, 53]. Stable binocular single vision is important for accurate spatial localization, depth perception, and efficient scanning of the driving environment. Diplopia may arise from orbital or neurologic conditions affecting the extraocular muscles (e.g., thyroid eye disease) or their neural control, including cranial nerve palsies and brainstem injuries; symptoms may vary with the direction of gaze and with viewing objects at various distances [54].

Direct epidemiologic evidence linking diplopia to crash risk is limited. However, simulator studies suggest that binocular visual disturbances may affect hazard detection and reaction time [55], while correction of ocular misalignment through strabismus surgery may improve sign recognition and driver confidence [56]. In addition to the direct impact on binocular vision, diplopia may produce visual confusion and increased cognitive load [57]. Clinical management may include prism correction, occlusion, or surgical treatment. People with diplopia are generally advised to avoid driving until symptoms are stabilized or corrected [57]. Vehicle ergonomics may also influence diplopic symptoms, as extreme mirror-gaze angles required in some vehicle configurations may provoke diplopia in individuals with ocular misalignment or gaze-dependent diplopia [52].

### Visuospatial and Perceptual Dysfunction

Visuospatial and perceptual processing are higher-order cortical visual functions that enable drivers to interpret complex visual scenes, allocation of spatial attention, and navigate dynamic traffic environments [58]. These functions are mediated by distributed cortical networks, particularly the dorsal occipito-parietal (“where”) pathway, which supports spatial awareness, motion perception, and visually guided action, and the ventral occipito-temporal (“what”) pathway, which enables object recognition and identification [59, 60].

Disruption of the “where” pathway may impair spatial attention and environmental awareness. A common clinical example is visuospatial neglect following stroke, in which individuals fail to attend to stimuli on one side of space, typically the left as result of right hemispheric stroke, despite intact visual fields [61]. More severe dysfunction of bilateral parieto-occipital networks, such as in Balint’s syndrome, may result in impaired visual attention (as result of simultanagnosia), difficulty shifting gaze (oculomotor apraxia), and deficits in visually guided behavior (optic ataxia) [62]. These impairments can compromise hazard detection and spatial navigation during driving.

In contrast, dysfunction of the “what” pathway may impair object recognition. Conditions such as visual object agnosia result in difficulty recognizing or identifying objects despite preserved visual acuity, which may affect interpretation of traffic signs or environmental cues. Neurodegenerative diseases more common in older adults, including posterior cortical atrophy and Parkinson’s disease, may affect these visuospatial and perceptual processes and contribute to driving impairment [63].

There is evidence from cohort and observational driving studies linking visuospatial dysfunction to negative driving outcomes. Performance on tests of visual processing speed and attention, particularly the Useful Field of View (UFOV) test, has been associated with increased crash involvement and safety-critical driving events among older adults [64]. The UFOV is a computerized test that measures the speed at which visual information can be processed, including the ability to detect and localize targets under divided and selective attention conditions, reflecting higher-order visual–cognitive processing relevant to everyday tasks such as driving [65]. Slower visual processing speed has also been linked to higher rates of at-fault crashes [27].

### Behavioral Compensation and Self-Regulation

Many older drivers with visual impairment modify their driving habits in response to perceived visual limitations through self-regulation, which includes avoiding challenging conditions such as nighttime driving, highways, heavy traffic, unfamiliar routes, or adverse weather, as well as reducing overall driving exposure [37]. Nighttime avoidance is often the earliest and most reported restriction, reflecting reduced night vision, glare intolerance, and decreased contrast sensitivity. Naturalistic driving studies show that visually impaired older drivers frequently limit exposure to visually demanding environments and prefer familiar or lower-complexity routes, while also adopting tactical compensatory behaviors such as slower driving speeds, increased following distance, and greater head and eye movements to enhance visual scanning [66, 67]. Environmental context further modifies risk, as visually demanding conditions, including low lighting, glare, complex intersections, high-speed traffic, and adverse weather, can increase driving difficulty [67], while roadway design, traffic density, and urban versus rural settings shape exposure to these high-risk situations [68].

## Clinical Evaluation of Driving in Older Adults with Visual Impairment

### Vision Screening in Licensing Systems

There is no universally accepted gold-standard visual screening tool for assessing driving fitness in older adults. Visual acuity remains the most commonly used test worldwide, typically requiring a minimum acuity of approximately 20/40 (6/12) in the better eye for an unrestricted private driver’s license [10, 34]. However, as discussed, the ability of visual acuity alone to predict driving safety remains limited, as driving performance depends on multiple visual domains that are not captured by standard acuity testing [20, 64]. Licensing policies vary substantially across jurisdictions. In the U.S., state-level regulations result in differences in screening requirements and renewal procedures; most states require ~ 20/40 acuity while some permit restricted driving with poorer acuity and others do not mandate routine vision screening at renewal [69]. Minimum requirements for degree of binocular peripheral vision vary even more widely. Similar variability exists internationally in screening age, reassessment frequency, and vision standards (Table 2), highlighting the lack of consensus on optimal approaches to evaluating visual fitness to drive.

**Table 2:** Vision Screening Requirements for Driver Licensing in Selected High-Income Countries

Region/Country	Visual Acuity Requirement	Other Vision Requirements	Age-Based Screening	Renewal Frequency	Key Notes
United States	~ 20/40 (6/12) (varies by state)	Variable; not uniformly required	Variable by state	Variable	State-level variation; some states do not require routine vision screening at renewal
Canada (Ontario)	~ 20/40	knowledge test	≥ 80 years	Every 2 years	Provincial regulation; Ontario shown here as representative example
Australia	20/40 (6/12)	Visual field and medical assessment when clinically indicated	Condition-based	Periodic	National guidelines (Austroads); ongoing review for progressive conditions
European Union	20/40 (6/12)	≥ 120° horizontal visual field	Variable	Variable	EU directive recommends standards; implementation varies by country, with some reliance on self-declaration
United Kingdom	Number plate test at 20 m (≈ 20/40)	None routinely required	≥ 70 years	Every 3 years	Self-declaration of visual fitness; no routine in-person vision testing
Japan	≥ 0.7 binocular (≈ 20/30)	No additional routine vision testing beyond acuity	≥ 75 years	At renewal	Mandatory cognitive screening at ≥ 75; additional testing if indicated
Singapore	Not uniformly specified	Vision assessment required for certain license classes (medical certification)	≥ 65 years	Periodic (varies by license class)	Shorter license validity with age; medical certification required for some drivers

Note: In countries where licensing is determined at subnational levels (e.g., United States, Canada), requirements may vary across jurisdictions. Ontario is shown as a representative example for Canada

### Clinical Assessment Beyond Visual Acuity

Visual functions other than high-contrast visual acuity, including visual field, contrast sensitivity, and visual processing speed, are more closely related to driving performance. No visual test reliably predicts driving safety when used in isolation [64], highlighting the need for a multimodal assessment interpreted within the broader clinical context, including medical history, neurologic status, and reported driving difficulties. In individuals with neurologic disease, such as stroke, Parkinson’s disease, or dementia, assessment of visuospatial and cognitive function is particularly important [15, 16]. When concerns persist, referral for functional driving evaluation may be appropriate. These assessments, often conducted by occupational therapists, combine off-road evaluation with on-road assessment in real traffic conditions. Driving simulators may also be used to assess hazard detection and reaction time under controlled conditions, although they may not fully replicate real-world driving environments.

## Management and Counseling of Drivers with Visual Impairment

Following clinical evaluation, management of driving safety in visually impaired older adults focuses on addressing reversible visual conditions, supporting adaptive strategies, and facilitating individualized decisions regarding continued driving. Treatment of modifiable ocular disease can significantly improve functional vision and driving outcomes. Cataract surgery, for example, has been associated with reduced motor vehicle crash risk, highlighting the importance of identifying and treating reversible causes of visual impairment [66]. Counseling should also address self-regulatory driving behaviors, as many older adults modify their driving habits in response to visual difficulties. When concerns about driving safety persist, referral for driver rehabilitation may provide structured assessment and training in compensatory strategies. For individuals with progressive visual or neurologic disease, early discussion of mobility planning and alternative transportation options can help facilitate safe transition while maintaining independence and quality of life.

Guidance from medical and regulatory bodies emphasizes a multidomain approach to driving assessment. The American Geriatrics Society recommends integrating visual, cognitive, and motor function and referral for comprehensive or on-road evaluation when concerns arise [70]. Similarly, neurologic guidelines, including those addressing Parkinson’s disease and dementia, highlight the importance of integrating cognitive, visuospatial, and motor assessments, along with periodic reassessment [71, 72, 73]. National standards, such as Austroads and the UK Driver and Vehicle Licensing Agency (DVLA), also conceptualize driving as a multidimensional task incorporating visual, cognitive, and functional components into fitness-to-drive evaluations [74, 75]. However, there remains no universally accepted vision-specific framework for assessing driving fitness, and current approaches rely on clinical judgment, functional assessment, and selected visual measures, without a standardized framework for integrating domain-specific visual findings into driving fitness decisions.

## Medicolegal and Ethical Considerations

Decisions regarding older drivers with visual impairment involve important ethical and legal considerations. For example, legal requirements regarding physician reporting of medically impaired drivers vary widely across states in the US. In some states reporting to licensing authorities is mandatory, whereas in others, it is discretionary. Clinicians should therefore be familiar with local regulations and professional guidelines and document counseling discussions carefully. Early, transparent communication about driving safety and mobility planning can help patients and families prepare for potential driving limitations. At the same time, for many older adults, driving represents mobility, social participation, and access to healthcare, recommendations to restrict or cease driving may have significant psychological and social consequences. Clinicians must balance respect for patient autonomy with their responsibility to promote public safety.

## Future Directions

Emerging research focuses on improving identification of drivers at risk through multidomain and technology-based assessment approaches. Digital risk prediction models integrating visual, cognitive, and demographic factors may provide more accurate estimates of driving safety than single-test screening. Advances in functional vision assessment, including digital visual field and contrast sensitivity measures and computerized cognitive–visual testing, may also improve clinical evaluation of driving ability. An important area for future research is the development of functionally relevant, sensory-adapted mobility measures that better capture how individuals with visual impairment interact with real-world driving environments. Such approaches may help bridge the gap between clinical testing and everyday driving performance. In-vehicle technologies, such as advanced driver-assistance systems (ADAS), including collision warnings and lane-departure alerts, may support hazard detection and improve driving safety for older adults with visual impairment [76, 77]. Looking ahead, semi-autonomous and autonomous vehicle technologies may offer new opportunities to preserve mobility, although their real-world effectiveness and accessibility remain to be determined [78].

## Conclusion

Driving safety in older adults with visual impairment is multifactorial, reflecting the interaction of visual, cognitive, and environmental factors. Although visual acuity thresholds remain the primary vision screening tool used in driver licensing systems worldwide, they provide only a limited measure of the visual abilities required for safe driving. Evidence suggests that impairments in other visual domains–such as peripheral vision, contrast sensitivity, and higher-order visual processing–may have greater impact on driving performance and crash risk. Counseling and management strategies should be individualized, emphasizing treatment of reversible visual conditions and referral for functional driving evaluation when appropriate. Continued research is needed to develop evidence-based screening approaches and policies that balance road safety with preservation of mobility and independence among older adults.

## Key References

Within last three years:

- [35] Nguyen H, Luca Di Tanna G, Coxon K, Brown J, Ren K, Ramke J, et al. Associations between vision impairment and driving performance and the effectiveness of vision-related interventions: A systematic review. Transportation Research Interdisciplinary Perspectives. 2023 Jan;17:100753. 10.1016/j.trip.2022.100753.
  - ○ This recent systematic review is highly relevant because it synthesizes evidence linking visual impairment with driving performance and reviews the effectiveness of vision-related interventions. It also highlights that multiple visual domains, rather than visual acuity alone, are relevant to driving safety.
- [71] Stamatelos P, Economou A, Yannis G, Stefanis L, Papageorgiou SG. Parkinson’s Disease and Driving Fitness: A Systematic Review of the Existing Guidelines. Mov Disord Clin Pract. 2024 Mar;11(3):198–208. 10.1002/mdc3.13942.
  - ○ This reference is particularly important because it systematically reviews existing guidance on driving fitness in Parkinson’s disease and shows that multidomain assessment, including motor, cognitive, visual, sleep, and medication-related factors, is emphasized, while specific test recommendations and cutoff values remain inconsistent.
- [78] Biassoni F, Gnerre M. Understanding Elderly Drivers’ Perception of Advanced Driver Assistance Systems: A Systematic Review of Perceived Risks, Trust, Ease of Use, and Usefulness. Geriatrics. 2024 Nov 5;9(6):144. 10.3390/geriatrics9060144.
  - ○ This systematic review is relevant because it examines factors influencing older adults’ acceptance and use of ADAS, including trust, perceived usefulness, safety, and ease of use. It is useful for understanding barriers to real-world adoption of these technologies in older drivers.
- [36] García-Lozada D, Rivera-Pinzón F, Ibáñez-Pinilla E. Relationship Between Visual Acuity, Colour Vision, Contrast Sensitivity and Stereopsis, and Road Traffic Accidents: A Systematic Review and Meta-Analysis. Safety. 2025 Jul 28;11(3):71. 10.3390/safety11030071.
  - ○ This recent meta-analysis is useful because it compares several visual domains in relation to road traffic accidents and shows that evidence is stronger and more consistent for some settings and driver groups than others. It also reinforces that visual acuity alone is an incomplete marker of driving risk.
- [18] Livingston G, Huntley J, Liu KY, Costafreda SG, Selbæk G, Alladi S, et al. Dementia prevention, intervention, and care: 2024 report of the Lancet Standing Commission. Lancet. 2024;404(10452):572–628. 10.1016/S0140-6736(24)01296-0.
  - ○ This report is relevant because the latest Lancet Standing Commission identifies visual impairment as a modifiable risk factor for dementia, reinforcing the broader neurologic and cognitive context in which visual dysfunction may affect driving safety in older adults.

## Data Availability

No datasets were generated or analysed during the current study.
